# Downregulation of osteoprotegerin in colorectal cancer cells promotes liver metastasis via activating tumor-associated macrophage

**DOI:** 10.1038/s41598-023-49312-w

**Published:** 2023-12-14

**Authors:** Wataru Hirata, Yoshiro Itatani, Hideyuki Masui, Kenji Kawada, Rei Mizuno, Takamasa Yamamoto, Takuya Okamoto, Ryotaro Ogawa, Susumu Inamoto, Hisatsugu Maekawa, Ryosuke Okamura, Yoshiyuki Kiyasu, Keita Hanada, Michio Okamoto, Yasuyo Nishikawa, Naoko Sugimoto, Takuya Tamura, Etsuro Hatano, Yoshiharu Sakai, Kazutaka Obama

**Affiliations:** 1 Department of Surgery, Graduate School of Medicine, Kyoto University, 54 Shogoin-Kawaharacho, Sakyo-ku, Kyoto, 606-8507 Japan; 2 Department of Surgery, Kurashiki Central Hospital, Okayama, 710-8602 Japan; 3 Department of Surgery, NHO Kyoto Medical Center, Kyoto, 611-0041 Japan; 4 Department of Surgery, Japanese Red Cross Osaka Hospital, Osaka, 543-8555 Japan; 5 Rogel Cancer Center, University of Michigan, Ann Arbor, MI 48109 USA

**Keywords:** Cancer, Gastroenterology, Oncology

## Abstract

Osteoprotegerin (OPG) is a secreted cytokine that functions as a decoy receptor for receptor activator of nuclear factor kappa-B (RANK) ligand (RANKL). Anti-RANKL treatment for bone metastasis has been widely accepted for solid tumors. However, the mechanism of OPG-RANKL-RANK signaling in systemic colorectal cancer (CRC) metastasis remains unclear. In this study, we investigated the relevance and function of OPG expression in CRC liver metastasis. First, we performed in silico analysis using The Cancer Genome Atlas public database and found that lower OPG expression in CRC was associated with poor overall survival. Immunohistochemistry analyses using resected specimen from patients with CRC in our institute confirmed the result. Patient-matched primary CRC and liver metastases showed a significant downregulation of OPG expression in metastatic lesions. In CRC cell lines, OPG expression did not suppress cell proliferation and migration. However, OPG expression inhibited macrophage migration by suppressing the RANKL-RANK pathway. Moreover, in vivo mouse liver metastasis models showed that OPG expression in CRC cells suppressed liver metastases. In addition, treatment with an anti-RANKL neutralizing antibody also suppressed liver metastases. These results showed that downregulation of OPG expression in CRC cells promotes liver metastasis by activating tumor-associated macrophage, which can become a candidate for targeted therapy with anti-RANKL neutralizing antibody for CRC liver metastasis.

## Introduction

Colorectal cancer (CRC) is one of the leading causes of cancer-related mortality in developed countries. Although most patients with CRC can be cured before metastases occur, once they metastasize to the distant organs, the 5-year survival rate decreases by approximately 15%^1^. Moreover, even after curative resection of CRC, approximately 15% suffer recurrence within 5 years, among which the liver is the most frequent, followed by the lung^2^. Therefore, it is necessary to develop treatment strategies for metastatic CRC.

The tumor microenvironment constitutes a complex network between tumor cells and the surrounding normal host cells comprised of immune cells, vascular endothelial cells, fibroblasts, and cells residing as normal organ tissues. The composition of the tumor microenvironment differs among organs, which may affect the therapeutic responses to chemotherapy^3^. It is well known that the tumor microenvironment contributes to tumor progression and resistance to chemotherapy for CRC^4–7^. In addition, the tumor microenvironment can also be a target for the treatment of advanced CRC with anticancer drugs, such as anti-angiogenic inhibitors and immune checkpoint inhibitors.

Osteoprotegerin (OPG), also known as tumor necrosis factor (TNF) receptor superfamily 11B (TNFRSF11B), is a secreted cytokine receptor that functions as a decoy receptor for receptor activator of nuclear factor kappa-B (RANK) ligand (RANKL) and TNF-related apoptosis-inducing ligand (TRAIL)^8–10^. OPG was first identified as a regulator of osteoclast differentiation and bone metabolism^8^. It has also been shown to play several cellular roles, including tumor growth and metastasis. Some types of tumor cells are known to metastasize to the bone, where OPG plays a critical role in skeletal metastasis formation in prostate, breast and lung cancers^11–13^. RANKL, the ligand of OPG, is abundantly expressed in the tumor stroma^14,15^, and tumor-associated macrophage (TAM) is one of the main players in the activated RANKL-RANK pathway in the tumor microenvironment^16^. However, the function of OPG-RANKL-RANK signaling in the tumor microenvironment of CRC has not yet been well investigated. In Fact, previous reports on the relationship between CRC prognosis and OPG expression have shown contradictory outcomes^17–19^. One study showed that reduced OPG expression due to promoter methylation was correlated with poor overall survival (OS)^17^. However, another report showed that mRNA expression of *OPG* was higher in patients with metastatic CRC, and that OPG protein overexpression was associated with poor OS and relapse-free survival (RFS)^19^. Therefore, the clinical significance of OPG expression in CRC cells remains controversial, and the molecular mechanisms by which OPG functions in CRC tissue to promote or suppress cancer cell progression is not fully understood.

The purpose of this study is to elucidate the molecular mechanisms of OPG in the tumor microenvironment of CRC, especially in liver metastasis, the most threatening condition of CRC, in the context of TAM, and to explore the potential of novel therapeutic strategies for CRC liver metastasis.

## Materials and methods

### Public database

Data from The Cancer Genome Atlas (TCGA) program related to CRC (colon adenocarcinoma [COAD] and rectal adenocarcinoma [READ]) were obtained, and patient survival was analyzed^20^. A total of 376 samples out of 736 enrolled in the COAD and READ database can be available for *OPG* expression from RNA-seq data in the primary tumor for survival analysis (Supplementary Fig. S1). The patient population was divided into two groups with a cut-off median value of OPG expression and Kaplan–Meier method was used to plot survival curves with survival time cutoff as 1826 days (5 years).

### Patients

A total of 198 patients with CRC who underwent primary resection at Kyoto University Hospital between June 2005 and December 2008 and 31 patients who underwent resection of liver metastases between June 2003 and December 2014 were retrospectively analyzed. The diagnosis of CRC was confirmed by pathological examination. The study protocol was approved by the Kyoto University Graduate School and Faculty of Medicine, Ethics Committee (approval number R-2908), and opt-out approach was used for their consents of the study.

### Cell lines and reagents

MC38 was kindly provided by Naoya Fujita (Japanese Foundation for Cancer Research)^21^. HCT116, SW480, HEK293T, and CMT93 were supplied from American Type Culture Collection (Manassas, VA, USA), and THP-1 was from RIKEN BRC (RCB1189). CRC cell lines and HEK293T were maintained in low glucose DMEM, and THP-1 was in RPMI 1640, with 10% fetal bovine serum (FBS) (MP Biomedicals, Solon, OH, USA) and 1% penicillin/streptomycin mixture (Wako, Osaka, Japan). For the preparation of conditioned medium to coculture THP-1 cells, cancer-cell conditioned medium was collected after 24 h incubation with 0.5% FBS in RPMI 1640, centrifuged for 5 min at 1000 rpm to remove cell debris, and filtered through 0.22 μm filter (Membrane Solutions, Auburn, WA, USA). Recombinant human soluble RANKL and denosumab were purchased from Peprotech (Cranbury, NJ, USA) and Daiichi Sankyo (Tokyo, Japan), respectively.

### THP-1 macrophage differentiation

THP-1 human monocytic leukemia cell line was differentiated into THP-1 macrophages (dTHP-1) by 72 h incubation with 100 nM phorbol 12-myristate 13-acetate (PMA) (Sigma-Aldrich, St. Louis, MO, USA), and rested in PMA-free medium for 24 h as described previously^22^.

### Osteoprotegerin knockout using CRISPR-Cas9 system

The oligonucleotides listed in Supplementary Table S1 were used to knock out human *OPG* and mouse *Opg* expression. Each set of oligonucleotides was annealed and cloned into the BbsI site of the pSpCas9(BB)-2A-GFP (px458) vector (Addgene, Cambridge, MA, USA). Sequences were confirmed using the hU6-F primer (Supplementary Fig. S3). SW480 or CMT93 cells were transfected with *OPG*-KO or *Opg*-KO plasmid using Lipofectamine 2000 (Invitrogen, Carlsbad, CA, USA), respectively, and single clones expressing GFP were sorted using FACSAria II. Both human *OPG*-KO and mouse *Opg*-KO were used to minimize off-target effects.

### Osteoprotegerin overexpression with lentiviral transduction

Human *OPG* and mouse *Opg* cDNA were cloned from SW480 and CMT93, respectively, using the oligonucleotides listed in Supplementary Table S1 by amplification with PrimeSTAR Max DNA polymerase (TAKARA Bio, Tokyo, Japan). The human *OPG* amplicon was cloned into BamHI and XhoI sites of pLEX-MCS (Addgene), and mouse *Opg* was cloned into SpeI and XhoI. The sequences were confirmed using the pLEX-MCS forward primer (Supplementary Table S1). Recombinant lentiviruses were generated by transient transfection of HEK293T cells with psPAX2, pMD2.G (Addgene), and pLEX using Lipofectamine 2000. For *OPG/Opg* overexpression (*OPG*-OE/*Opg*-OE), HCT116 or MC38 cells were transduced with the *OPG*-OE or *Opg*-OE lentivirus and selected with puromycin (1.0 μg/mL) as a pool to minimize clonal variation.

### Western blotting

Cells were lysed in NP-40 lysis buffer (50 mmol/L Tris–HCl, pH 8.0, 150 mmol/L NaCl, 10% glycerol, 1% NP-40), and protease inhibitor or phosphatase inhibitor [Nacalai Tesque, Kyoto, Japan]. Cell lysates were subjected to sodium dodecyl sulfate polyacrylamide gel electrophoresis, and transferred to Immobilon P membranes (Millipore, Billerica, MA). The membranes were blocked with Blocking One (Nacalai Tesque, Kyoto, Japan) for 20 min at room temperature and then immunoblotted with the primary antibodies at 4 ℃ overnight followed by horseradish peroxidase-conjugated secondary antibodies at room temperature for 30 min. β-Actin was used as a loading control.

### Quantitative reverse transcription polymerase chain reaction (qRT-PCR)

Total RNA was extracted using the High Pure RNA extraction kit (Roche) according to the manufacturer’s protocol. Complementary DNA generated by reverse transcription was quantified using StepOnePlus Real Time PCR system (Applied Biosystems). The primer probe sets were listed in Supplementary Table S2. The mRNA levels were normalized to that for *ACTB* by a ΔΔCt method.

### Enzyme-linked immunosorbent assay (ELISA)

OPG protein levels were measured with ELISA kits (Human OPG DuoSet DY805 and Mouse Opg Duoset DY459, R&D Systems), according to manufacturer’s protocol. CRC cell lysates were extracted in NP-40 lysis buffer as described above. Conditioned medium of CRC cell lines was prepared by seeding 1.0 × 10^6^ cells for human CRC cell lines and 5.0 × 10^5^ cells for mouse CRC cell lines in 6-well plates with 1 mL medium for 24 h. The absorbance was measured at 450 nm using the plate reader GLOMAX-Multi + Detection System (Promega, Madison, WI, USA).

### Immunohistochemistry (IHC) and immunofluoresence (IF)

For IHC, formalin-fixed, paraffin-embedded (FFPE) sections were stained with anti-OPG (NOVUS, 98A1071, [1:1000]), anti-CD68 antibody (Dako, PG-M1, [1:200]), anti-CD206 antibody (Cell Signaling Technology, E2L9N, [1:400]), or anti-mouse CD8 (eBioscience, 4SM15, [1:100]) using the avidin–biotin immunoperoxidase method. The expression of OPG was evaluated by assessing cytoplasmic staining and graded as negative (−), weak (+), moderate (++) or strong (+++) as described previously, by two researchers (W. H. and Y. I.) independently without prior knowledge of clinical data^17,19,23^. No heterogenous staining was observed withing individual slides and estimation of the proportion of staining was not required as described previously^17^. Slides with different evaluations among the two researchers were interpreted once again, followed by a conclusive judgement, and divided into 2 groups; low-expression with negative and weak staining and high-expression with moderate and strong staining as described previously^17,19^. The CD68 and CD206 positive area proportion was evaluated by selecting five non-overlapped fields and the staining area per total tissue area of visual field (× 400) using Hybrid Cell Count (BZ-X800 analyzer, KEYENCE, Osaka, Japan). Similarly, the number of CD8 positive cells were counted by selecting five non-overlapping fields (× 400). For IF, FFPE human sections or 4% paraformaldehyde-fixed mouse cryosections were incubated with anti-CD68, anti-CD206, anti-RANK (Santa Cruz, H-7, [1:50]), anti-mouse F4/80 (eBioscience, BM8, [1:200]) or anti-mouse CD206 (Bio-Rad, MR5D3, [1:100]) followed by incubation with a secondary antibody.

### Transwell migration assay

Migration of dTHP-1 cells was assessed using 24-well Transwell cell culture chambers (5 μm-pore membrane) (CORNING, Corning, NY, USA). A total of 1.0 × 10^5^ THP-1 cells were placed in the upper chamber, differentiated, and rested as described above. After starvation overnight with 0.5% FBS, medium with 100 ng/ml RANKL alone or RANKL plus 30 µg/ml denosumab was applied, or cancer cells with/without OPG expression/knockout were seeded and co-cultured, and stimulated with RANKL. After incubation for 24 h, non-migrated cells were removed from the upper surface of the membrane using a wet cotton swab, and cells on the lower surface of the membrane were stained with 0.5% crystal violet. The migrated cells were counted in five fields (× 400).

### In vivo mouse models

Animal study was approved by Animal Research Committee, Graduate School of Medicine, Kyoto University (approval number Med Kyo 22158). All methods were carried out in accordance with relevant guidelines and regulations, and all studies are reported in accordance with ARRIVE guidelines (https://arriveguidelines.org). For experimental liver metastasis models, 5.0 × 10^6^ HCT116 cells or 1.0 × 10^6^ MC38 cells were injected into the spleen of 8-week-old female Balb/cSlc-nu/nu nude mice (for HCT116 xenograft model) or syngeneic wild-type C57BL/6 J mice (for MC38 allograft model) under general anesthesia with isoflurane. For treatment with the anti-Rankl antibody (Mab clone IK22/5 or isotype rat IgG2 antibody, BioXCell), mice were treated intraperitoneally at a dose of 5.0 mg/kg once every other day for 3 weeks, and euthanized at 11-week of age to measure the liver weights and representative section for assessment of tumor burden.

### Histomorphometric analysis

Formalin-fixed, paraffin-embedded sections of representative mouse livers were stained with hematoxylin and eosin. Tumor area and normal liver area were evaluated using Hybrid Cell Count (BZ-X800 analyzer, KEYENCE, Osaka, Japan), (Supplementary Figs. S8 and S9).

### Statistical analysis

All values are expressed as mean ± standard deviation (SD). The statistical significance of differences was determined by student’s *t* test, Mann–Whitney *U* test or chi-square test. All analyses were 2-sided, and a *P* value of < 0.05 was considered statistically significant. Statistical analyses were performed using JMP Pro software version 14.0 (SAS Institute, Cary, NC, USA). For Kaplan–Meier estimate curves, we used Prism8 software (MDF, Tokyo, Japan).

## Results

### Downregulation of OPG expression in CRC was associated with poor survival

**Figure 1:**
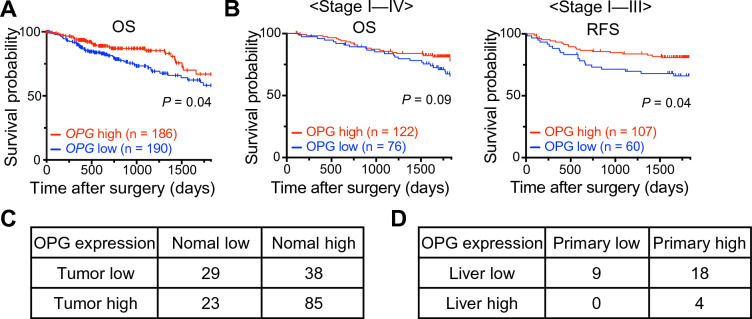
Expression of OPG and survival of patients with CRC. (**A**) Kaplan–Meier estimates between *OPG*-high and -low (cut-off value is median value) in patients with COAD and READ from TCGA (376 cases, *P* = 0.04, log-rank test). (**B**) Kaplan–Meier estimates between OPG-high and -low in patients who underwent primary CRC resection at our institute. The left panel shows the OS in patients with Stage I–IV disease (198 cases, *P* = 0.09, log-rank test), and the right panel shows the RFS in Stage I–III disease (167 cases, *P* = 0.04, log-rank test). (**C**) Patient-matched OPG expression between normal intestine and colon cancer tissues (175 cases, *P* < 0.01, chi-square test). (**D**) Patient-matched primary CRC and liver metastasis who underwent both resection at our institute (31 cases).

To elucidate the role of OPG in CRC, we first investigated the relationship between the *OPG* expression levels and prognosis using TCGA database (COAD and READ) (Fig. 1A and Supplementary Fig. S1). The Kaplan–Meier estimates of 376 patients with 5-year survival showed that lower *OPG* expression was significantly correlated with poor OS, as assessed by the log-rank test (*P* = 0.04). To confirm this result obtained from TCGA database, we also examined resected specimens of human CRC at our institute by IHC and assessed them as described previously (Supplementary Fig. S2A)^19^. Patient characteristics were similar between the low and high OPG expression groups, except for tumor location (Table 1). Primary CRC specimens obtained from 198 continuous patients showed that low OPG expression was correlated with poor OS (198 patients, stages I–IV, *P* = 0.09, log-rank test) and RFS (167 patients, stages I–III, *P* = 0.04, log-rank test) (Fig. 1B). We also compared OPG expression between normal and tumor colonic tissues in 175 of the 198 patients for whom normal intestinal tissues were available. We found that OPG expression was significantly lower in tumor tissues than in normal intestinal tissues (*P* < 0.01, chi-square test) (Fig. 1C). Given that lower OPG expression was significantly correlated with poor RFS in our cohort, and that the liver is the most frequent site of recurrence in CRC, we then compared OPG expression between the primary site and liver metastases obtained from 31 patients with CRC who showed liver metastases and underwent resection at both sites at our institute (Fig. 1D, Supplementary Fig. S2B and Table 2). CRC liver metastases with low OPG at the primary site were associated with more metachronous recurrences and more recurrences after liver resection than those with high OPG (Table 2). All patients who showed low OPG expression at the primary site also showed low OPG expression in the liver metastases. However, many patients with high OPG expression at the primary site lost expression when they metastasized to the liver.

**Table 1:** Univariate analysis of clinicopathologic factors of primary resection (n = 198).

Variables	OPG expression		*P*
	Low (n = 76)	High (n = 122)	
Age, y			0.66
Mean ± SD	67.4 ± 11.1	66.7 ± 10.8	
Gender			0.81
Male	48 (63.2)	75 (61.5)	
Female	28 (36.8)	47 (38.5)	
Location			< 0.05
Colon	53 (69.7)	62 (50.8)	
Rectum	23 (30.3)	60 (49.2)	
Histology			0.52
tub1/tub2	67 (88.2)	111 (91.0)	
por/muc	9 (11.8)	11 (9.0)	
T factor			0.36
Tis/T1/T2	15 (19.7)	31 (25.4)	
T3/T4	61 (80.3)	91 (74.6)	
N factor			0.25
Negative	48 (63.2)	67 (54.9)	
Positive	28 (36.8)	55 (45.1)	
M factor			0.1
Negative	60 (79.0)	107 (87.7)	
Positive	16 (21.0)	15 (12.3)	
Stage			0.48
0–II	45 (59.2)	66 (54.1)	
III–IV	31 (40.8)	56 (45.9)	
Lymphatic invasion			0.17
Negative	36 (47.4)	70 (57.4)	
Positive	40 (52.6)	52 (42.6)	
Venous invasion			0.89
Negative	31 (40.8)	51 (41.8)	
Positive	45 (59.2)	71 (58.2)	
CEA			0.06
< 5	37 (48.7)	76 (62.3)	
≥ 5	39 (51.3)	46 (37.7)	
CA19-9			0.13
< 37.0	59 (77.6)	105 (86.1)	
≥ 37.0	17 (22.4)	17 (13.9)	

Characteristics of patients divided according to OPG status. Chi-square test and Student’s t test were used for categorical items and continuous variables, respectively.

**Table 2:** Univariate analysis of clinicopathologic factors of liver resection (n = 31).

Variables	OPG expression of primary CRC		*P*
	Low (n = 9)	High (n = 22)	
Age, y			0.79
Mean ± SD	66.3 ± 10.1	65.2 ± 10.3	
Gender			0.68
Male	5 (55.6)	14 (63.6)	
Female	4 (44.4)	8 (36.4)	
Location			0.88
Right side colon (C/A/T)	2 (22.2)	4 (18.2)	
Left side colon (D/S/RS)	6 (66.7)	14 (63.6)	
Rectum (Ra/Rb/P)	1 (11.1)	4 (18.2)	
Histology			0.07
tub1/tub2	8 (88.9)	22 (100.0)	
por/muc	1 (11.1)	0 (0.0)	
T factor			0.86
Tis/T1/T2	1 (11.1)	2 (9.1)	
T3/T4	8 (88.9)	20 (90.9)	
N factor			0.32
Negative	2 (22.2)	9 (40.9)	
Positive	7 (77.8)	13 (59.1)	
Chronology			< 0.05
synchronous	4 (44.4)	19 (86.4)	
metachronous	5 (55.6)	3 (13.6)	
Recurrence after liver ressection			0.04
No	1 (11.1)	11 (50.0)	
Yes	8 (88.9)	11 (50.0)	

Characteristics of patients divided according to OPG status of primary CRC**.** Chi-square test and Student’s t test were used for categorical items and continuous variables, respectively.

### OPG expression does not affect CRC proliferation or migration

**Figure 2:**
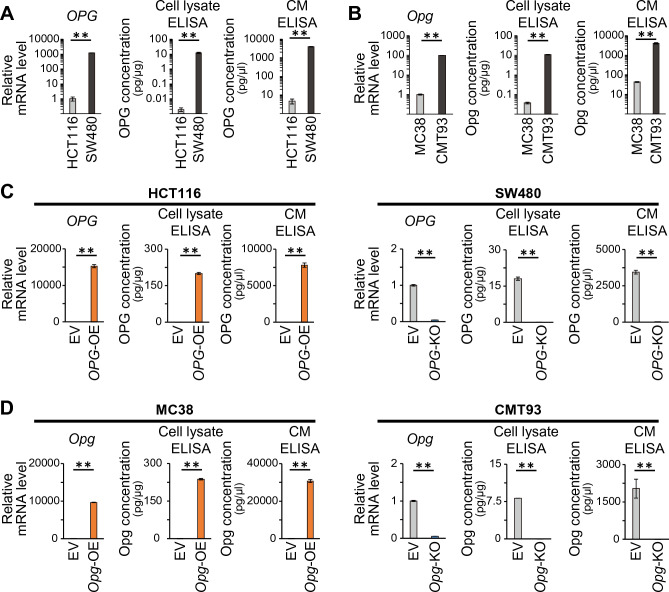
OPG expression in human and mouse CRC cell lines. (**A**, **B**) qRT-PCR and ELISA analyses showing OPG expression levels of human (**A**) and mouse (**B**) CRC cell lines. CM indicates conditioned medium. Bars, mean ± SD, *n* = 3. (**C**) qRT-PCR and ELISA analyses showing OPG expression when OPG was overexpressed in HCT116 or knocked out in SW480. EV, OE, and KO indicate empty vector, overexpression, and knockout, respectively. Bars, mean ± SD, *n* = 3. (**D**) qRT-PCR and ELISA analyses showing Opg expression when Opg was overexpressed in MC38 or knocked out in CMT93. Bars, mean ± SD, *n* = 3. (**, *P* < 0.01).

To investigate the role of OPG expression in CRC cells, we first examined the expression levels of human OPG/mouse Opg in CRC cell lines. qRT-PCR analysis revealed that OPG/Opg was almost undetectable in HCT116 and MC38, whereas SW480 and CMT93 expressed high levels of OPG (Fig. 2A,B). Quantitative protein expression assessed by ELISA using the cell lysates and cancer cell-conditioned medium from each cell line confirmed these results. We generated OPG/Opg-overexpressing cell lines by lentiviral transduction using HCT116 and MC38 and *OPG*/*Opg*-knockout cell lines by CRISPR-Cas9 gene editing using SW480 and CMT93, and confirmed mRNA and protein expression levels by ELISA (Fig. 2C,D, and Supplementary Fig. S3). Neither OPG/Opg overexpression nor knockout affected cancer cell proliferation, as assessed by CCK8 cell proliferation assay and cell count assay (Supplementary Figs. S4A and S4B). The scratch wound-healing assay also showed no significance in cancer cell migration, regardless of whether OPG/Opg was overexpressed or knocked out in CRC cells (Supplementary Figs. S4C and S4D).

### OPG expression suppresses macrophage activation via blocking RANKL-RANK signaling

**Figure 3:**
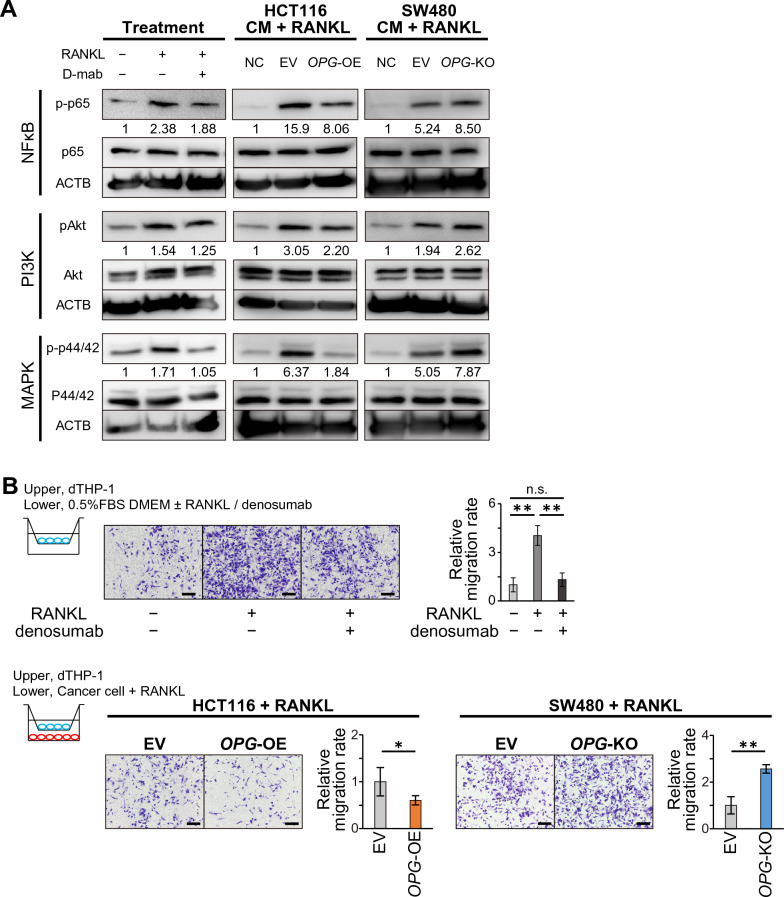
Macrophage activation via the OPG-RANKL-RANK pathway. (**A**) Western blot and densitometry readings/intensity ratio showing phosphorylation status of RANK pathway when treated with RANKL, denosumab or cancer cell conditioned medium plus RANKL (upper panels, NFκB; middle panels, PI3K; lower panels, MAPK pathways). NC indicates no-cell medium, and EV indicates conditioned medium from cells with empty vector. The samples derive from the same experiment and that blots were processed in parallel. Original blots are presented in Supplementary Fig. S4. Bars, mean ± SD, *n* = 3. (**B**) Transwell migration assay of dTHP-1 when treated with RANKL or RANKL plus denosumab or co-culture with cancer cells plus RANKL. Bar graphs show relative areas of migrated dTHP-1 cells in five random fields. Scale bar, 200 μm. Bars, mean ± SD, *n* = 5.

Given that the downregulation of OPG in CRC had no direct effect on CRC proliferation or migration, we then assessed the correlation between CRC and TAM, one of the major players in the tumor microenvironment with respect to the RANKL-RANK pathway. THP-1 is a human monocyte leukemia cell line that can be differentiated into macrophages using phorbol 12-myristate 13-acetate (PMA), which is widely accepted for in vitro macrophage experiments^24^. We confirmed that PMA treatment promoted THP-1 differentiation (dTHP-1) to express M2 macrophage markers and higher levels of RANK and RANKL (Supplementary Figs. S5A, S5B and S5C)^25,26^. The major activation pathways of the RANKL-RANK axis include the nuclear factor kappa B (NFκB), phosphatidylinositol-3 kinase (PI3K) and mitogen-activated protein kinase (MAPK) pathways^27^. As expected, dTHP-1 cells responded to RANKL stimulation in the downstream NFκB, PI3K, and MAPK signaling pathways, which can be suppressed by the anti-RANKL neutralizing antibody denosumab (Fig. 3A, Supplementary Figs. S6 and S7). We then treated dTHP-1 cells with conditioned media from HCT116 or SW480 cells. Conditioned medium from OPG-expressing HCT116 cells (HCT116-OPG) suppressed these pathways in dTHP-1, which was activated by the medium from control cells (HCT116-EV) (Fig. 3A Supplementary Figs. S6 and S7). In contrast, conditioned medium from SW480 cells without OPG expression (SW480 *OPG*-KO) promoted the activation of these pathways in dTHP-1 cells compared to that in control cells (SW480-EV) (Fig. 3A, Supplementary Figs. S6 and S7). We also assessed macrophage activity using a Transwell migration assay with dTHP-1 cells. As expected, RANKL stimulation activated dTHP-1 migration, which was suppressed by anti-RANKL denosumab administration (Fig. 3B). To mimic the tumor microenvironment, we stimulated dTHP-1 cells with RANKL, and seeded CRC cells in the lower chamber. Higher OPG expression from CRC cells (HCT116-OPG or SW480-EV) suppressed the migration of activated dTHP-1 cells, whereas a low or no OPG environment (HCT116-EV or SW480 *OPG*-KO) promoted dTHP-1 migration (Fig. 3B).

**Figure 4:**
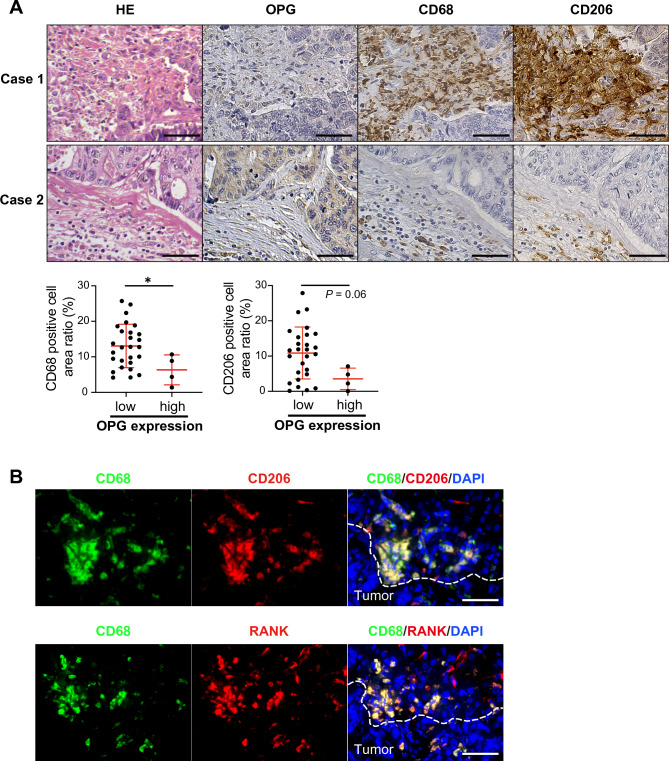
IHC and IF analysis of CRC liver metastasis (n = 27 and 4 in low and high groups, respectively). (**A**) IHC analyses of OPG, CD68 and CD206 in CRC liver metastasis. Scale bar, 50 μm. (**B**) Representative pictures of IF in CRC liver metastasis with CD68, CD206 and RANK. Scale bar, 50 μm. (*, *P* < 0.05).

Based on the results of the migration assay, we attempted to confirm the relationship between OPG expression and TAM accumulation in the clinical specimens of CRC liver metastases. IHC analyses showed that the CD68-positive (macrophage marker) area was significantly larger in the liver metastases with low OPG expression, with the same trend as that of the CD206-positive (M2 phenotype macrophage marker) area (Fig. 4A). Furthermore, IF revealed that CD68-positive cells were also positive for CD206 and RANK, indicating M2 phenotype macrophages expressing RANK (Fig. 4B).

### Anti-RANKL treatment is a favorable targeted therapy for OPG-deficient CRC liver metastases

**Figure 5:**
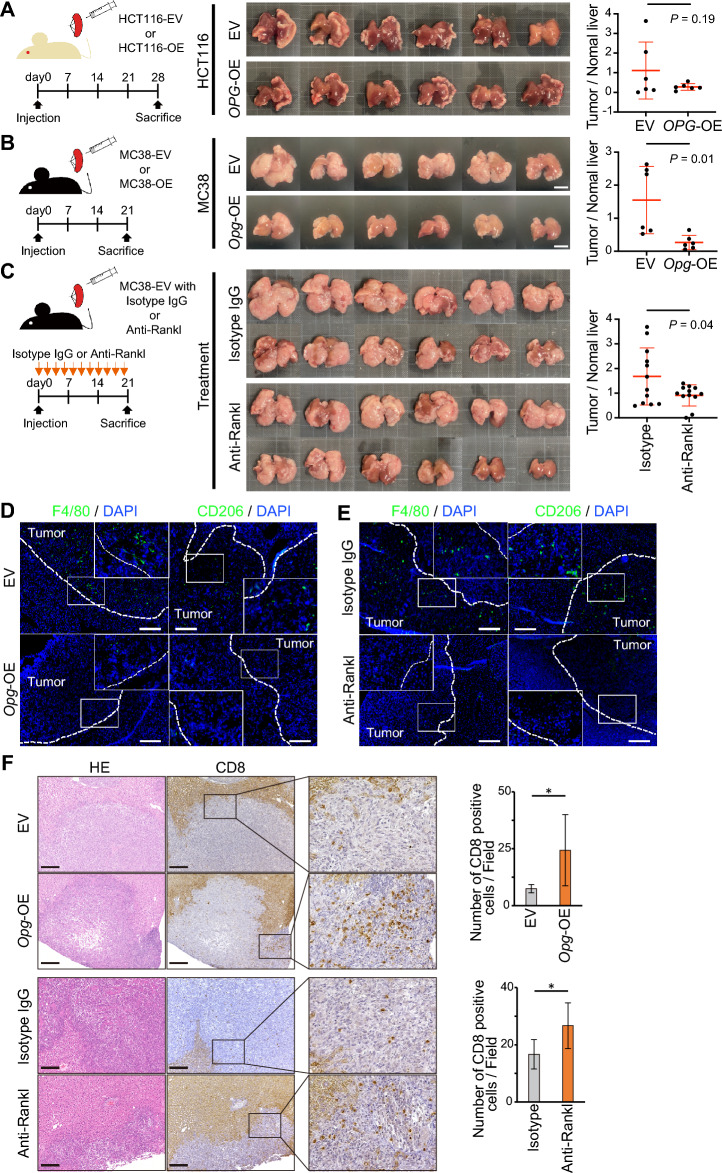
Mouse xenograft and allograft models of CRC liver metastasis with or without OPG expression. (**A**, **B**) Quantification of liver metastasis by normal liver area of the xenograft model with HCT116 (**A**) or allograft model with MC38 (**B**) when cancer cells expressing or not expressing OPG were injected into the spleen and sacrificed at day 21 or 28. N = 6 in each group. (**C**) Quantification of liver metastasis by normal liver area of MC38 allograft model treated with or without the anti-Rankl neutralizing antibody. N = 12 in each group. (**D**, **E**) Representative pictures of metastatic liver foci from MC38 cells with Opg overexpression (**D**) and anti-Rankl treatment (**E**) stained with F4/80 or CD206. Scale bar, 200 μm. (**F**) Representative pictures of CD8 IHC from metastatic liver foci of MC38 allograft models and quantification of CD8^+^ cells per field at the metastases. Scale bar, 200 μm. Bars, mean ± SD. (*, *P* < 0.05).

As described above, downregulation of OPG expression in CRC cells may promote liver metastasis by activating TAM, resulting in poor prognosis. To confirm that downregulation of OPG expression in patients with CRC can be used as a biomarker for targeted treatment, we investigated the effect of OPG expression in the mouse models of CRC liver metastasis. We used OPG-deficient cell lines (HCT116 and MC38), overexpressing OPG/Opg, and created liver metastases. Both xenograft HCT116-OPG and allograft MC38-Opg cells showed lower liver/body weight ratios than HCT116-EV and MC38-EV cells, respectively, suggesting that OPG expression can suppress liver metastasis (Fig. 5A,B, Supplementary Fig. S8). Histomorphometric analyses showed a significant suppression of MC38-Opg metastatic areas over MC38-EV, but a marginal difference between HCT116-OPG and HCT116-EV metastatic areas (Fig. 5A,B, Supplementary Figs. S8 and S9). Importantly, histomorphometric analyses showed that anti-mouse Rankl neutralizing antibody treatment with MC38-EV significantly suppressed liver metastasis, with the same trend for the liver/body weight ratio (Fig. 5C, Supplementary Fig. S8), suggesting that anti-RANKL treatment can be a favorable targeted therapy against OPG-negative CRC liver metastasis. In addition, IF revealed that OPG overexpression or anti-Rankl treatment suppressed TAM accumulation in the liver metastases (Fig. 5D,E), resulting in the significant infiltration of CD8^+^ killer T-cells (Fig. 5F) in the tumor microenvironment.

## Discussion

Previous reports have shown contradictory results regarding the relationship between CRC prognosis and OPG expression^17–19^, and the clinical significance of OPG expression in CRC cells remains controversial. To this end, we first performed in silico analysis by using TCGA public database, and found that downregulation of *OPG* expression was associated with poor OS (Fig. 1A). IHC analyses using resected specimens from our institute confirmed that OPG was significantly downregulated in CRC compared to normal colonic tissue, and downregulation of OPG expression was significantly correlated with poor OS in CRC (Fig. 1B,C). In addition, metastatic liver tumors from CRC showed significant downregulation of OPG compared with primary CRC (Fig. 1D and Table 2). Taken together, downregulation of OPG can promote malignant progression and metastasis of CRC to the liver. Next, we addressed the molecular mechanism by which downregulation of OPG promotes cancer cell progression. In vitro analyses have suggested that OPG expression does not affect cancer cell proliferation or migration in an autocrine manner (supplementary Fig. S4). Instead, OPG expression from CRC cells suppresses M2 macrophage activity by inhibiting NFκB, PI3K, and MAPK signaling pathways, the major RANKL-RANK pathways (Fig. 3A,B). In vivo analyses showed that low OPG expression promoted CRC liver metastasis by recruiting CD206-positive TAM to the liver microenvironment, and that the RANKL-RANK pathway blockade with OPG overexpression or anti-RANKL antibody suppressed CRC liver metastasis by inhibiting TAM accumulation (Figs. 4 and 5). Taken altogether, downregulation of OPG expression promotes CRC liver metastasis, and it can be a surrogate marker for anti-RANKL treatment of CRC liver metastasis. To our knowledge, this is the first study to elucidate the molecular mechanism by which OPG downregulation promotes CRC metastasis and to show that blockade of this pathway can be a favorable treatment strategy for CRC liver metastasis.

The OPG-RANKL-RANK pathway plays a critical role in bone metastasis, and the anti-RANKL neutralizing antibody denosumab is a standard treatment for bone metastases from solid tumors to inhibit osteoclast activity and reduce skeletal-related events^28^. Osteoclasts, monocyte/macrophage lineage cells in skeletal tissue, promote bone metastases through the NFκB, PI3K, and MAPK signaling pathway by RANKL stimulation^29–32^. The RANKL-RANK axis is also involved in various stages of cancer progression^33–36^ and can be neutralized by OPG functioning as a decoy receptor. Our results indicate that denosumab treatment and OPG overexpression suppressed dTHP-1 activity by inhibiting these signaling pathways through which TAM can be polarized to the M2 (pro-tumoral) phenotype to contribute to tumor progression^37–41^.

Recently, there has been emerging interest in targeting tumor microenvironment for cancer treatment. TAM is one of the main players in the tumor microenvironment and have not yet been clinically used to specifically target TAM. Macrophages in the tumor microenvironment contain heterogeneous populations that are schematically known as M1 (anti-tumoral) and M2 phenotypes^42^. In vitro analyses showed that dTHP-1 expressed M2 macrophage markers, and that downregulation of OPG in CRC promoted dTHP-1 migration by activating RANKL-RANK pathway (Fig. 3A,B, Supplementary Figs. S5A and S5B). It has been now accepted that TAM is mainly composed of the M2 phenotype, and our IHC results also confirmed that the CD68-positive macrophages in the tumor microenvironment were also positive for CD206, suggesting that they are of the M2 phenotype (Fig. 4B). M2 macrophages have a pro-tumoral effect by suppressing tumor-infiltrating lymphocytes such as CD8^+^ killer T-cells^16^. In this study, we did not observe sufficient suppression of liver metastasis from HCT116-OPG cells in a xenograft model using nude mice, probably because the mice lacked CD8^+^ T-cells. M2 macrophages become expressing RANK in certain environments to promote tumor progression^43^, which was also observed in our study (Fig. 4B).

The present study elucidates the molecular mechanism by which OPG downregulation in CRC cells promotes liver metastasis in relation to TAM in the tumor microenvironment. OPG expression is downregulated in various cancer cell lines, including colon cancer^44^. Therefore, the downregulation of OPG can be a favorable biomarker for CRC liver metastasis, and the inhibition of the RANKL-RANK pathway by OPG or anti-RANKL antibodies in such patients can provide a new therapeutic strategy for CRC liver metastasis. The limitation of this study is that we mainly performed in vitro and in vivo mouse experiments. Therefore, further clinical studies are required to confirm the therapeutic effects of anti-RANKL antibodies against a subgroup of CRC liver metastases with low OPG expression.

### Supplementary Information

Supplementary Information.

## Data Availability

The TCGA datasets are available online. The other datasets in our institute used in this study are available from the corresponding authors on reasonable request.
