# Supplementary material for: Downregulation of osteoprotegerin in colorectal cancer cells promotes liver metastasis via activating tumor-associated macrophage
Source: Sci Rep. 2023 Dec 14;13:22217. doi: 10.1038/s41598-023-49312-w (PMC10721637; doi:10.1038/s41598-023-49312-w)
Supplement: Supplementary file 1 — Supplementary Information. [file 41598_2023_49312_MOESM1_ESM.docx]

**Downregulation of osteoprotegerin in colorectal cancer cells promotes liver metastasis via activating tumor-associated macrophage**

Supplementary information

**Supplementary Figures**

**Figure S1.** Flowchart of sample selection for TCGA COAD and READ database. There were 376 samples whose sample types were primary tumors and included OPG expression. Four samples were excluded from OPG-High group due to the lack of the survival data.

**Figure S2.** IHC of OPG

1. Representative pictures of primary CRC specimens. Left two panels show low OPG expression (no or week OPG expression) and right two panels show high OPG expression (moderate or strong OPG expression) in CRC cells. Scale bar, 100 μm.
2. Representative pictures of patient-matched primary CRC and liver metastasis who underwent both resection at our institute (31 cases). Scale bar, 100 μm.

**Figure S3.** Sequencing analyses of CRISPR/Cas9-mediated *OPG/Opg* knockout.

1. Nucleotide sequence chromatogram of SW480 EV/*OPG*-KO. Sequence analysis revealed that both alleles had one nucleotide insertion in *OPG* gene.
2. Nucleotide sequence chromatogram of CMT93 EV/*Opg*-KO. Sequence analysis revealed that one allele had one nucleotide insertion and the other had 2 nucleotides deletion in *Opg* gene.

**Figure S4.** Cell proliferation and migration with or without OPG expression.

(A, B) CCK8 and cell count assays with or without OPG expression in human (A) and mouse (B) cell lines. Bars, mean ± SD, n = 3.

(C, D) Scratch wound-healing assay with or without OPG expression in human (C) and mouse (D) cell lines. Bar graphs indicate the relative width of the wound. Bars, mean ± SD, n = 3. (n.s., no significance).

**Figure S5.** THP-1 differentiation by PMA stimulation.

1. Expression of indicated macrophage markers in dTHP-1 assessed by qRT-PCR analysis. Bars, mean ± SD, *n* = 3.
2. Expression of M2 macrophage markers in dTHP-1 assessed by qRT-PCR analysis. Bars, mean ± SD, *n* = 3.
3. Expression of RANK and RANKL in dTHP-1 assessed by qRT-PCR analysis. Bars, mean ± SD, *n* = 3.

**Figure S6.** The second and the third sets of western blot analyses among triplicate experiment showing phosphorylation status of RANK pathway when treated with RANKL, denosumab or cancer cell conditioned medium related to Figure 3A.

Original western blot images for Figure 3A

Original western blot images for Figure 3A

Original western blot images for Figure S6A

Original western blot images for Figure S6A

Original western blot images for Figure S6B

Original western blot images for Figure S6B

**]**

**Figure S7.** These images are the original western blot images for Figure 3A, Supplementary Figure S6A, and S6B. Some membranes were cut prior to hybridization with antibodies to save the reagents.

**Figure S8.** Histomorphometric analysis of sections of mouse liver and quantification of the liver metastasis. (A, B, C) Sections of representative livers of the xenograft model with HCT116 (A) or allograft model with MC38 (B) or MC38 allograft model treated with or without the anti-Rankl antibody (C), were stained with hematoxylin and eosin. Orange areas indicate metastatic nodules. (D, E) Quantification of liver metastasis by liver volume of the xenograft model with HCT116 EV/OPG-OE (D) or allograft model with MC38 EV/Opg-OE (E). N = 6 in each group. (F) Quantification of liver metastasis by liver volume of MC38 allograft model treated with or without the anti-Rankl antibody. N = 12 in each group.

**Figure S9.** Higher magnification of Figure S8. S9A, S9B and S9C correspond to S8A, S8B and S8C, respectively.

**Supplementary Table S1. List of primer sequences.**

| human *OPG*#1-sense | 5’-CACCGGCAGTATAGACACTCGTCAC-3’ |
| --- | --- |
| human *OPG*#1-anti-sense | 5’-AAACGTGACGAGTGTCTATACTGCC-3’ |
| mouse *Opg*#1-sense | 5’-CACCGAACAGCACTGCACAGTGAGG-3’ |
| mouse *Opg*#1-anti-sense | 5’-AAACCCTCACTGTGCAGTGCTGTTC-3’ |
| hU6-F | 5’-GAGGGCCTATTTCCCATGATT-3’ |
| human *OPG*-forward | 5’-AGCAAAGTGGAAGACCGTGT-3’ |
| human *OPG*-reverse | 5’-AGGGCAGCTCCTATGTTTCA-3’ |
| mouse *Opg*-forward | 5’-AATGTGCTCCTGGCACCTAC-3’ |
| mouse *Opg*-reverse | 5’-TAACGCCCTTCCTCACACTC-3’ |
| pLEX-MCS forward | 5’-CACCAAAATCAACGGGACTT-3’ |

**Supplementary Table S2. List of primer probe sets for qRT-PCR.**

| human *TNFSF11* | Applied Biosystems, Hs00243522_m1 |
| --- | --- |
| human *TNFRSF11A* | Applied Biosystems, Hs00921372_m1 |
| human *TNFRSF11B* | Applied Biosystems, Hs00900358_m1 |
| mouse *Tnfrsf11b* | Applied Biosystems, Mm00435454_m1 |
| human *ACTB* | Applied Biosystems, HS99999903_m1 |
| mouse *Actb* | Applied Biosystems, Mm00607939_s1 |
| human *CD11b* | Forward; 5’-GGA ACG CCA TTG TCT GCT TTC G-3’  Reverse; 5’-ATG CTG AGG TCA TCC TGG CAG A-3’ |
| human *CD68* | Forward; 5’-CGA GCA TCA TTC TTT CAC CAG CT-3’  Reverse; 5’-ATG AGA GGC AGC AAG ATG GAC C-3’ |
| human *CD14* | Forward; 5’-CTG GAA CAG GTG CCT AAA GGA C-3’  Reverse; 5’-GTC CAG TGT CAG GTT ATC CAC C-3’ |
| human *CD36* | Forward; 5’-CAG GTC AAC CTA TTG GTC AAG CC-3’  Reverse; 5’-GCC TTC TCA TCA CCA ATG GTC C-3’ |
| human *CD71* | Forward; 5’-ATC GGT TGG TGC CAC TGA ATG G-3’  Reverse; 5’-ACA ACA GTG GGC TGG CAG AAA C-3’ |
| human *TGFB* | Forward; 5’-TAC CTG AAC CCG TGT TGC TCT C-3’  Reverse; 5’-GTT GCT GAG GTA TCG CCA GGA A-3’ |
| human *IL10* | Forward; 5’-TCT CCG AGA TGC CTT CAG CAG A-3’  Reverse; 5’-TCA GAC AAG GCT TGG CAA CCC A-3’ |
| human *CD206* | Forward; 5’-AGC CAA CAC CAG CTC CTC AAG A-3’  Reverse; 5’-CAA AAC GCT CGC GCA TTG TCC A-3’ |
| human *ACTB* | Forward; 5’-GCA AAG ACC TGT ACG CCA AC-3’  Reverse; 5’-ACA TCT GCT GGA AGG TGG AC-3’ |
